# Relationship between actinic keratosis and malignant skin lesions on the eyelid

**DOI:** 10.5935/0004-2749.20230002

**Published:** 2023

**Authors:** Luiz Angelo Rossato, Rachel Camargo Carneiro, Erick Marcet Santiago de Macedo, Patricia Picciarelli de Lima, Mariana Ragassi Urbano, Suzana Matayoshi

**Affiliations:** 1 Post-Doctorate Program in Ophthalmology, Department of Ophthalmology, Universidade de São Paulo, São Paulo, SP, Brazil.; 2 Department of Ophthalmology, Universidade de São Paulo, São Paulo, SP, Brazil.; 3 Department of Pathology and Ophthalmology, Universidade de São Paulo, São Paulo, SP, Brazil.; 4 Department of Statistics, Universidade Estadual de Londrina, Londrina, PR, Brazil.

**Keywords:** Keratosis, actinic/pathology, Biopsy, Eyelid neoplasms, Eyelids/injuries, Ceratose actínica/patologia, Biópsia, Neoplasmas palpebrais, Pálpebra/lesões

## Abstract

**Purpose:**

To evaluate the variables possibly related to actinic keratosis and malignant skin lesions on the eyelid.

**Methods:**

A prospective study of patients with suspected eyelid malignancy was conducted. The participants underwent a 2-mm punch biopsy at two opposite sites of the lesion for diagnosis, and the results were compared with those of the histopathological study of the surgical excised specimen. The patients with an actinic keratosis component were divided into two groups (actinic keratosis-associated malignancy and actinic keratosis alone), which were compared for the following variables: age, disease duration, largest diameter, tumor area, Fitzpatrick classification, sex, tumor site and margin involvement. A cluster analysis was also performed.

**Results:**

We analyzed 174 lesions, of which 50 had an actinic keratosis component. Actinic keratosis was associated with squamous cell carcinoma in 22% of the cases and to basal cell carcinoma in 38%, which shows that both neoplasms may have contiguous actinic keratosis. Statistical analysis revealed no significant difference among the variables. In a cluster analysis, four groups were identified with malignant lesions in the medial canthus with the largest mean diameter and area. All margin involvements on the lower eyelid were related to malignancy, which means that all cases with margin involvement had an almost 100% risk of malignancy.

**Conclusions:**

Larger actinic keratosis lesions in the medial canthus and lesions with margin involvement on the lower eyelid have a greater probability of malignant association.

## Abstract (pt)

**Objetivo:**

Avaliar as possíveis variáveis relacionadas à ceratose actínica e lesões malignas cutâneas nas pálpebras.

**Métodos:**

Estudo prospectivo de pacientes com lesões palpebrais suspeitas de malignidade. Os participantes foram submetidos à biopsia por trépano (punch) de 2-mm em dois pontos opostos da lesão como método diagnóstico e os resultados foram comparados com o estudo histopatológico da peça excisada cirurgicamente. Aqueles que apresentaram ceratose actínica como resultado foram divididos em dois grupos (ceratose actínica associada com malignidade e ceratose actínica isolada) e foram comparados de acordo com as variáveis: idade, tempo de doença, maior diâmetro, área do tumor, classificação de Fitzpatrick, gênero, localização e acometimento da margem palpebral. A análise de cluster também foi realizada.

**Resultados:**

Foram analisadas 174 lesões e 50 delas tinham ceratose actínica como componente do tumor. Ceratose actínica esteve associada ao Carcinoma Espinocelular em 22% dos casos e ao Carcinoma Basocelular em 38%, mostrando que ambos podem ter ceratose actínica adjacente. A análise estatística não encontrou diferença entre as variáveis. A análise de cluster identificou quatro grupos e mostrou que lesões malignas no canto medial tinham maiores diâmetro e área. Acometimento da margem na pálpebra inferior relacionou-se em 100% com malignidade, enquanto a ausência de acometimento da margem mostrou menor chance de malignidade.

**Conclusões:**

Lesões maiores de ceratose actínica no canto medial e lesões com acometimento da margem palpebral inferior têm maiores chances de associação com malignidade.

## INTRODUCTION

Actinic keratosis (AK), senile keratosis, or solar keratosis is a benign and chronic photo-induced cutaneous lesion frequently observed in adults. It occurs in sun-damaged areas of the skin and is one of the signs of skin aging^(1)^. The prevalence is higher in men (up to 34%) and increases with age^(2,3)^. AK is an intraepithelial neoplasm formed by atypical differentiation and proliferation of keratinocytes, mostly induced by ultraviolet radiation^(2)^. Most lesions are slow-growing papules or plaques, <1 cm in diameter, dry, erythematous, pigmented with telangiectasias, and frequently covered with adherent scales and occur in chronically sun-exposed sites^(2)^.

As AK and squamous cell carcinoma (SCC) have similar genetic expression profiles^(3)^, untreated AK may develop into SCC in some patients, and AK is also a risk marker of basal cell carcinoma (BCC) and melanoma^(2)^, any confirmed or suspected lesion requires close follow-up. Lesions that appear clinically active must be investigated and treated^(3)^.

We examined consecutive patients with suspected eyelid malignancy, performed a 2-mm punch biopsy, and then compared the results with those of the histopathological study of the surgical specimen excised with clear margins by performing a frozen section analysis. In this study, we analyzed all the results with an AK component to establish possible relationships between eyelid AK and malignant lesions and to determine the efficacy of the 2-mm punch biopsy as a diagnostic method for achieving better diagnosis, treatment, and follow-up.

## METHODS

We examined consecutive patients with suspected eyelid malignancy who visited the oculoplastic service of the Department of Ophthalmology, Hospital das Clínicas, Medical School of the University of São Paulo (HC-FMUSP), between March 2019 and March 2020. The institutional research ethics board approved the study protocol under the entry No. 3.212.238. All the participants signed the written informed consent form.

We included patients with biomicroscopically suspected eyelid malignancy based on the following findings: changes in skin texture, color, pigmentation, and size associated with ulceration, elevated surface, irregular outline, telangiectasias, or loss of eyelashes. The exclusion criteria were as follows: patients with previously known diagnosis, recurrence, or lesions of <4 mm in diameter.

Previously, we documented all lesions with a high-resolution digital camera (Sony DSC-W125, Sony Corporation, Tokyo, Japan) mounted on a tripod and then measured the tumor area and largest diameter with the ImageJ 1.44 software (National Institute of Mental Health, Bethesda, Maryland, USA)^(4)^.

**Figure 1:**
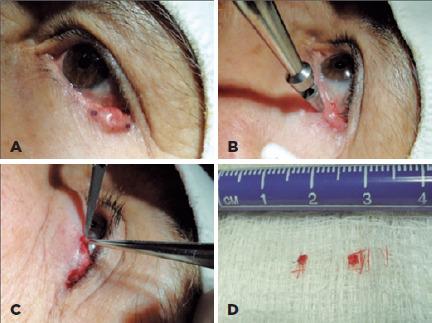
Two-site 2-mm punch biopsy of a lesion on the lower eyelid. (A) Markings indicating biopsy sites of the lesion on each extremity. (B) Boring into the tumor with a 2-mm punch. (C) Removal of samples using a forceps and No. 11 scalpel. (D) Collected specimens.

The patients underwent the standard 2-mm punch biopsy at a typical tumor site, which was performed by the same ophthalmologist, at the HC-FMUSP outpatient surgery center. Two specimens were taken from opposite extremities of the lesion, corresponding to the largest diameter (Figure 1). Within 15 to 60 days, if the biopsy diagnosis was malignant tumor or AK, the lesion was excised, with clear margins in the frozen section analysis, followed by eyelid reconstruction with the most appropriate technique for each case. The same pathologist (PPL), who was blinded to previous punch biopsy results, examined all the histopathological specimens at the Department of Pathology (HC-FMUSP).

The histological pattern of the lesion was determined on the basis of its growth examined with hematoxylin and eosin staining observed under ×200, ×400, and ×1000 (immersion) magnification, as needed. The World Health Organization criteria were used to classify the tumors, and mixed tumors were classified according to predominant (present in >50% of the samples) and secondary patterns^(5,6)^.

To determine the efficacy of the 2-mm punch biopsy for correct diagnosis of AK and possible association with malignancy, biopsy findings were compared with the histopathological examination of the complete surgical excised specimen. Then, lesions were divided into two groups (AK with associated malignancy and AK alone, at biopsy or at surgical excision of specimens). To compare the variables among the groups, the quantitative variables (age, disease duration, largest diameter, and tumor area) were analyzed using the *t* test or Wilcoxon test, while qualitative variables (Fitzpatrick classification, gender, tumor site, and eyelid margin involvement) were analyzed using the Fisher exact test. The level of statistical significance was set at 5%.

To determine the correct diagnosis of AK without malignancy by performing a 2-mm punch biopsy, the sensitivity, specificity, positive predictive value (PPV), negative predictive value (NPV), overall accuracy, and Kappa coefficient were calculated.

Cluster analysis was also performed with group variables divided into homogeneous clusters to identify the differences between them. The cluster analysis maximized the similarity of the cases within each cluster and the dissimilarity between the groups. Cluster variables were selected according to the level of importance, which ranged from 0 to 1. When the level of importance is zero or close to zero, the variable should not be included in the clusters, as it is not a relevant variable in clustering data. However, a variable at level 1 or approximately 1 must be included in the clusters^(7)^. The number of clusters was defined in accordance with the silhouette criterion measures of cohesion and separation, which range from −1 and 1. Values >0.5 indicate high-quality clusters^(8)^. All the analyses were performed with the R Core Team 2020 software^(9)^.

## RESULTS

To achieve our study objective, we analyzed 174 lesions, of which 50 had an AK component or were alone or associated with neoplasms at biopsy or surgery, as shown in table 1.

**Table 1:** Distribution of 50 lesions, according to the result of punch biopsy and surgical specimens

Punch biopsy	Surgical specimen	Number of lesions
AK	AK	20
AK	AK and BCC	1
AK	BCC	11
AK	AK and SCC	1
AK	SCC	9
AK and BCC	AK and BCC	2
AK and BCC	BCC	4
AK and BCC	SCC	1
BCC	AK	1
**TOTAL**		**50**

AK= actinic keratosis; BCC= basal cell carcinoma; SCC= squamous cell carcinoma.

The mean age of the 50 patients with an AK component was 67.18 years. Of the patients, half were female and half were male. The mean disease duration was 2.30 years, and the mean diameter and area of the lesion were 10.30 mm and 65.09 mm^2^, respectively. As for the skin phototypes, 88% of the patients had Fitzpatrick skin types 1-3. The most common site was the lower eyelid (84%). Of the sites, the upper eyelid and medial canthus corresponded to 8% each.

Statistical analysis revealed that both the quantitative and qualitative variables had no significant differences between the two groups (AK with associated malignancy and AK alone). The p values for age, disease duration, largest diameter, and tumor area were 0.39, 0.88, 0.63, and 0.23, respectively. As for the qualitative variables, the p value was 0.70 for the Fitzpatrick classification, 0.77 for gender, 0.28 for tumor site, and 0.57 for eyelid margin involvement.

As for the correct diagnosis of isolated AK by the 2-mm punch biopsy at two sites, the sensitivity was 95.24% and specificity was 24.14%, with an accuracy of 54% (95% confidence interval: 0.3932-0.6819) and Kappa coefficient of 0.1703. The PPV and NPV were 47.62% and 87.5%, respectively. AK was associated with SCC in 22% of the cases and to BCC in 38%, indicating that both neoplasms may have contiguous AK. In the cluster analysis, four clusters were identified, with a silhouette criterion measure of cohesion and separation of 0.6. The results are presented in table 2, including the comparison of the variables among the clusters for the qualitative variables (the Fisher exact test followed by a *z* test to compare the percentages among the groups) and quantitative variables (analysis of variance followed by the Tukey test to compare the means among the groups).

**Table 2:** Results of cluster analysis including the variables of association with malignancy, tumor site, margin involvement, mean tumor area and diameter, and variables comparison among the clusters

Cluster n (%)	Cluster 1 17 (34.0%)	Cluster 2 11 (22.0%)	Cluster 3 5 (10.0%)	Cluster 4 17 (34.0%)	
Variable					p-value
**Malignancy**					<0.01*
**Yes** n (%)	8 (47.1%) a***	0 (0.0%) b	5 (100.0%) ac	17 (100.0%) c	
**No** n (%)	9 (52.9%) a	11 (100.0%) b	0 (0.0%) ac	0 (0.0%) c	
**Tumor site**					<0.01*
**Lower eyelid** n(%)	14 (82.4%) abc	10 (90.9%) c	1 (20.0%) b	17 (100.0%) ac	
**Upper eyelid** n(%)	3 (17.6%) a	1 (9.1%) a	0 (0.0%) a	0 (0.0%) a	
**Medial canthus** n(%)	0 (0.0%) a	0 (0.0%) a	4 (80.0%) b	0 (0.0%) a	
**Margin**					<0.01*
**Yes** n(%)	0 (0.0%) a	11 (100.0%) b	2 (40.0%) c	17 (100.0%) b	
**No** n(%)	17 (100.0%) a	0 (0.0%) b	3 (60.0%) c	0 (0.0%) b	
**Largest diameter**; mean (SD)	9.56 (4.58) a	8.92 (2.57) a	21.29 (8.21) b	8.71 (3.19) a	<0.01**
**Tumor area**; mean (SD)	52.83 (58.43) a	32.10 (17.06) a	254.14 (186.49) b	43.09 (32.33) a	<0.01**

*p-value obtained in the Fisher Exact test followed by the z-test.

**p-value obtained in the ANOVA followed by the Tukey test.

***Equal letters indicate there are no differences between the percentages nor means among the clusters, and different letters indicate there are such differences.

The association with malignancy presented a higher percentage in cluster 4 than in clusters 1 and 2 and did not differ from the percentage in cluster 3. No associated malignancy presented a higher percentage in cluster 2 than in the other clusters, and no significant differences in percentages were found between clusters 1 and 3, and clusters 3 and 4.

For the variable tumor site, the lower eyelid presented higher percentages in clusters 2 and 4 than in cluster 3, and the percentage in cluster 1 did not significantly differ from the percentages in clusters 2, 3, and 4. The upper eyelid does not present differences in percentages between the clusters, whereas the medial canthus presented a higher percentage in cluster 3 than in the other clusters.

For margin involvement, clusters 2 and 4 presented higher percentages than clusters 1 and 3, and cluster 3 also presented a higher percentage than cluster 1. For no margin involvement, cluster 1 presented a higher percentage than the other clusters, and cluster 3 also presented a higher percentage than clusters 2 and 4. The largest diameter and tumor area presented higher mean values in cluster 3 than in the other clusters.

## DISCUSSION

AK, senile keratosis, or solar keratosis is a benign and chronic photo-induced cutaneous lesion frequently observed in adults aged ≥40 years. The prevalence is higher in men (up to 34%) and increases with age. Our study shows equal distribution between men and women, but the ages (mean, 67.18 years) and Fitzpatrick classifications (1-3 phototypes at 88% of the cases) were similar to those reported in the literature^(1,2,3,10)^.

AK is an intraepithelial neoplasm formed by atypical differentiation and proliferation of keratinocytes, mostly induced by ultraviolet radiation^(2,11)^. Therefore, chronic sun exposure and sunburns are the most important risk factors. The secondary risk factors are old age, male sex, place of birth with a higher ultraviolet radiation index, Caucasian ethnicity, history of previous skin neoplasms, organ transplantation, immunosuppression, outdoor occupation, and light phototypes (according to the Fitzpatrick classification)^(12,13,14,15,16)^.

Some characteristics of AK indicate that it should be treated as soon as it is diagnosed. The combinations of predisposing factors to AK, BCC, and SCC are similar. AK and SCC have similar genetic expression profiles, and AK is also a risk marker of BCC and melanoma. AK has a cumulative risk of 5%-20% for developing into SCC and similar rates of AK progression to BCCe. Untreated AK may develop into SCC in 8%-20% of the patients, and 27%-82% of SCCs are estimated to evolve from previous AK. Despite that 87%-97% of SCCs have contiguous AK, no specific clinical features can predict the AK lesions that would progress to neoplasms. For all these reasons, AK is considered a premalignant lesion^(2,3,10,11,13,16,17,18,19)^.

Twelve months after treatment, 25%-75% of patients may require retreatment depending on the previous treatment. Recurrence may result from incomplete elimination of the lesion, progression of subclinical lesions to clinical status, and development of new lesions^(13,20)^.

**Figure 2:**
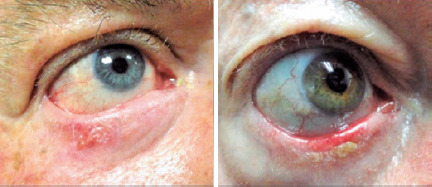
Two different cases of AK on the lower eyelid.

AK constitutes an important cause for medical consultation with dermatologists (second in the United States of America and fourth in Brazil)^(16)^. Patients with AK usually have multiple lesions, and the sites more chronically exposed to the sun are the face, neck, chest, dorsum of the hands, shoulders, and scalp. Most lesions are slow-growing papules or plaques, <1 cm in diameter, dry, erythematous, pigmented with telangiectasias, and frequently covered with adherent scales (Figure 2). Secondary ulceration may be quite variable^(2,10,14,16)^.

On the basis of all these features, the aim of this study was to identify a better diagnostic method for eyelid AK and possible relationships between AK and neoplasms.

Owing to the difficult clinical and definitive diagnosis prior to treatment, we chose the 2-mm punch at two sites of the lesion as the biopsy method because it is a quick, simple, and sutureless procedure that shows a high level of agreement with the traditional incisional biopsy^(21)^. When performed at two sites, the 2-mm punch biopsy allowed greater accuracy for the diagnosis of aggressive BCC subtypes than when performed at one site^(22)^.

In our AK series, the 2-mm punch biopsy, even when performed at two different sites of the tumor, showed low rates of accuracy (54%), specificity (24.14%), and PPV (47.61%). Most cases had contiguous neoplasms at biopsy or surgery. Therefore, we could infer that the 2-mm punch biopsy is not the ideal method for the diagnosis of eyelid AK or that AK is a lesion associated with neoplasms.

As AK is confined within the epidermis, it is usually a more peripheral lesion when associated with neoplasms. The epidermis directly contiguous or adjacent to a SCC shows evidence of AK, and an invasive carcinoma is usually found in deeper sections of the lesions initially diagnosed as AK by biopsy. Thus, an incisional biopsy that does not reach deeper tissues might not provide a correct diagnosis^(23)^. This could clarify why some malignant tumors were not shown in the 2-mm punch biopsy. Further studies are necessary to find a better diagnostic method for AK on the eyelid.

In spite of these poor diagnostic indicators in the 2-mm punch biopsy, we found that some AK patterns on the eyelid that could guide the treatment decision and made identification of these patterns our main study objective. Although the quantitative and qualitative variables analyzed had no significant differences between the two groups, cluster analysis revealed features of possible malignant relationship. In cluster 3, we found that most of the malignant lesions were in the medial canthus and had the largest mean diameters and areas. Comparing clusters 1 and 4, we found that the margin involvement on the lower eyelid was related to a 100% risk of malignancy (cluster 4), while no margin involvement had an almost 50% risk of malignancy (cluster 1). Thus, larger AK lesions in the medial canthus and lesions with margin involvement on the lower eyelid have a greater probability of malignant association. López-Tizón et al. suggested that AK lesions with margin involvement might behave more aggressively and more easily progress to SCC than those located away from the margin^(18)^, which was proven by our data.

Our series had a higher rate of AK relationship with BCC (38%) than with SCC (22%), unlike most cases in the dermatology literature(^10,15,16,24^). As we analyzed only eyelid AK, we could infer that neoplasms may have a different behavior at the periocular skin because of its peculiarities and specialized adnexa.

Therefore, AK remains difficult to diagnose. All lesions suspected of AK must be investigated, treated, and closely followed up, particularly those on the eyelid, for which the main choice of treatment is surgery excision with safety margins (because topical agents may cause local reaction and eyelid retraction)^(18)^. Functional and cosmetic treatment results are of great implications, with potential impact on patient quality of life^(16)^. Herein lies the importance of examiner experience in the diagnosis and treatment of the disease. In this study, we found evidence that can guide oculoplastic surgeons in the management of suspected AK lesions on the eyelid, especially those in the medial canthus and on the lower eyelid, for a more precocious follow-up and definitive treatment.
